# Supplementary material for: Transcriptomics-based drug repositioning pipeline identifies therapeutic candidates for COVID-19
Source: Res Sq. 2021 Mar 30:rs.3.rs-333578. Preprint. [Version 1] doi: 10.21203/rs.3.rs-333578/v1 (PMC8020993; doi:10.21203/rs.3.rs-333578/v1)
Supplement: Supplement [file 0a22132ca97b81db353f6d1e.pdf]

Supplementary Materials

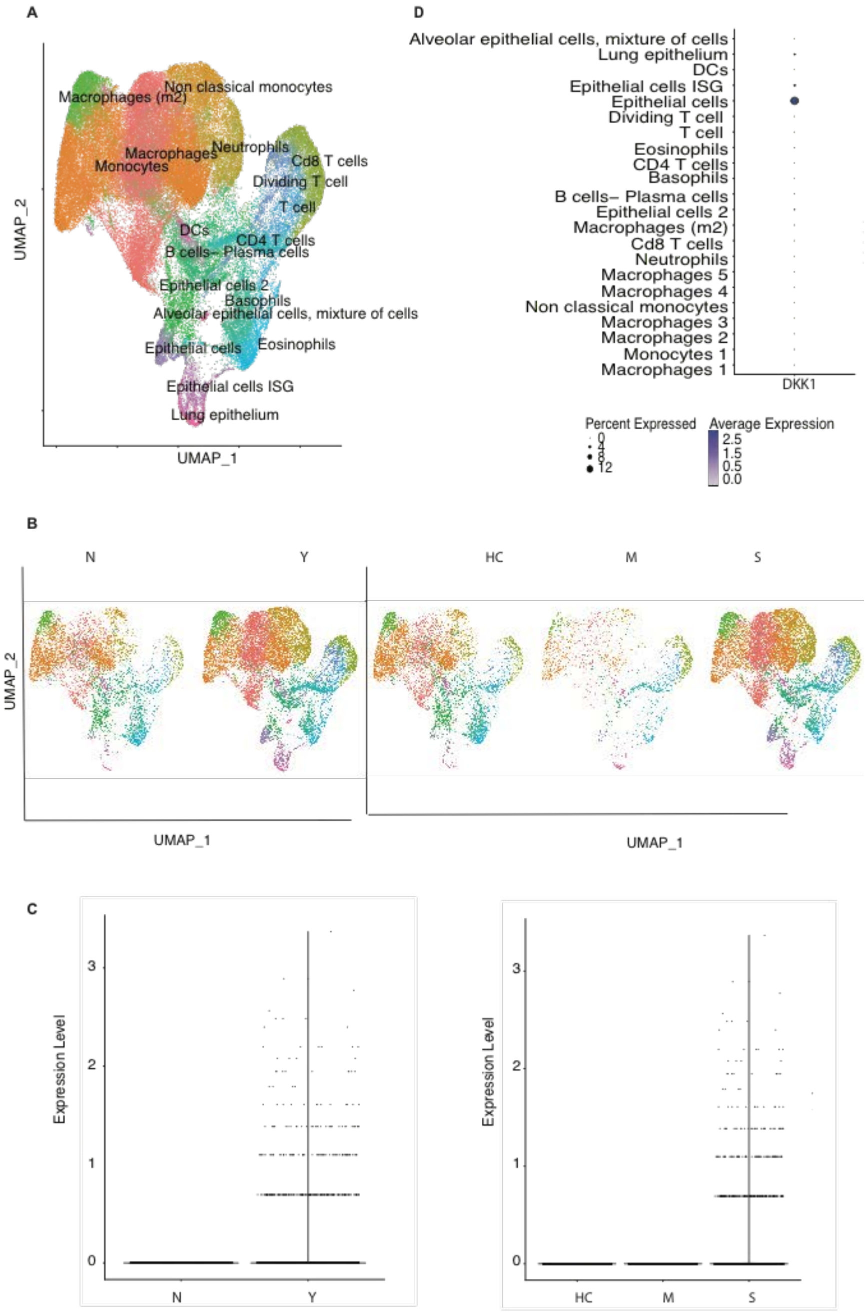

Figure S1. Figure S1. We checked the expression of *DKK1* in the GSE128033 dataset composed of 4 healthy (HC), 3 presenting with mild COVID-19 symptoms (M), and 6 presenting with severe COVID-19 symptoms (S). In our analyses, **(A)** we observed 21 clusters, with **(B)** no batch effect. **(C)** DKK1 seems to be expressed in COVID-19 patients (Y = COVID-19 patients, N = healthy). **(D)** DKK1 is exclusively expressed in epithelial cells.

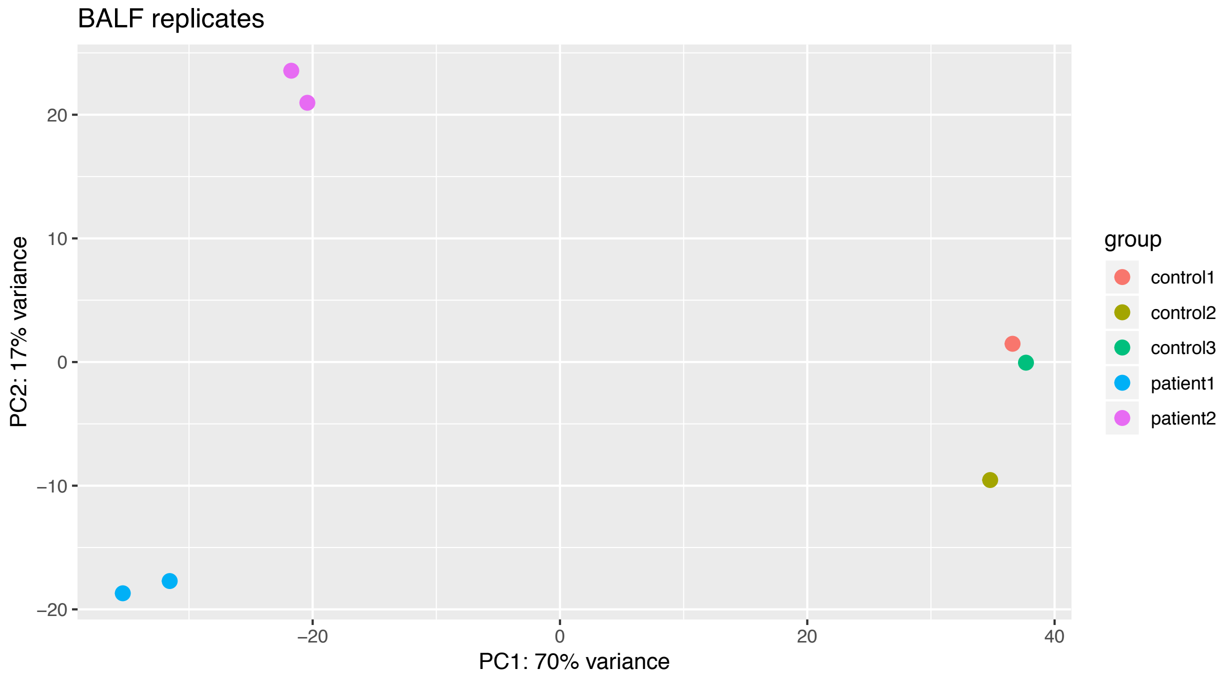

Figure S2. PCA of BALF signature.

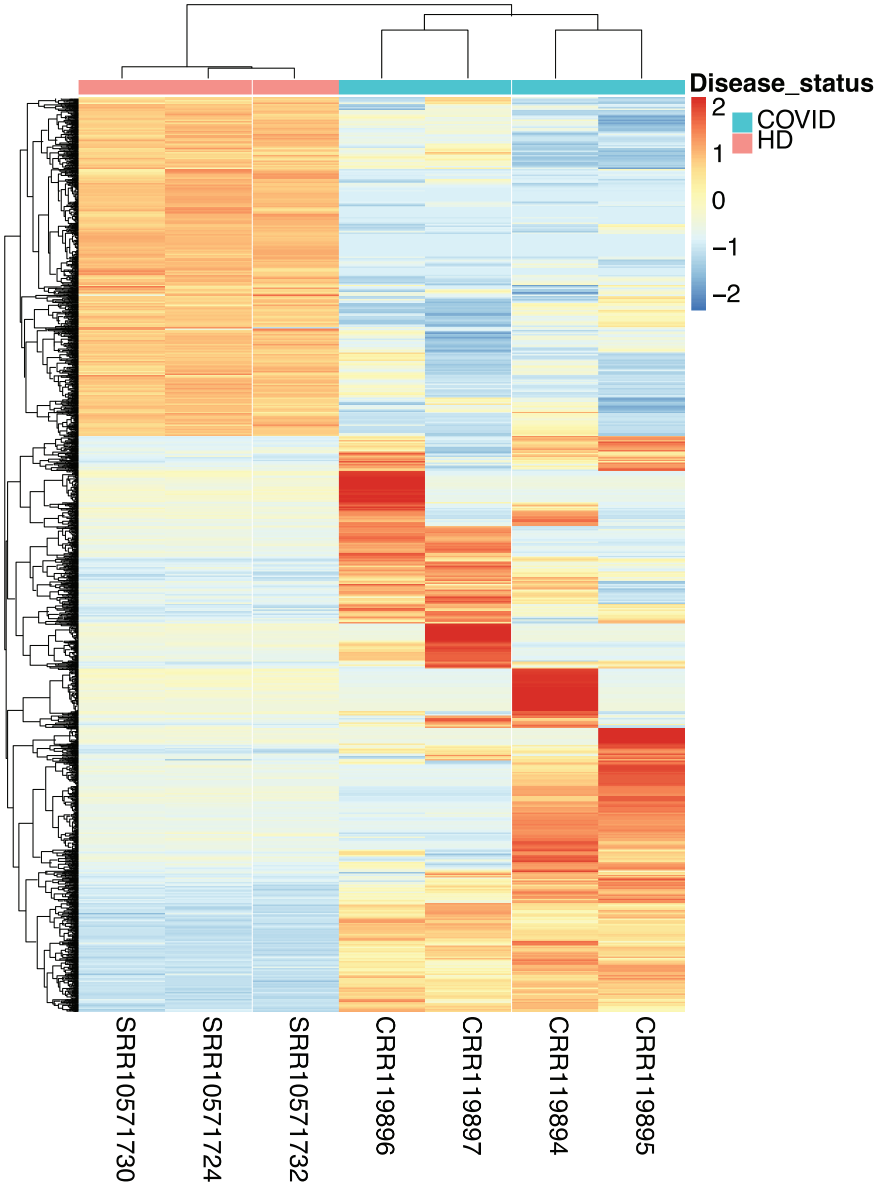

Figure S3. Heatmap of gene expression from BALF signature.

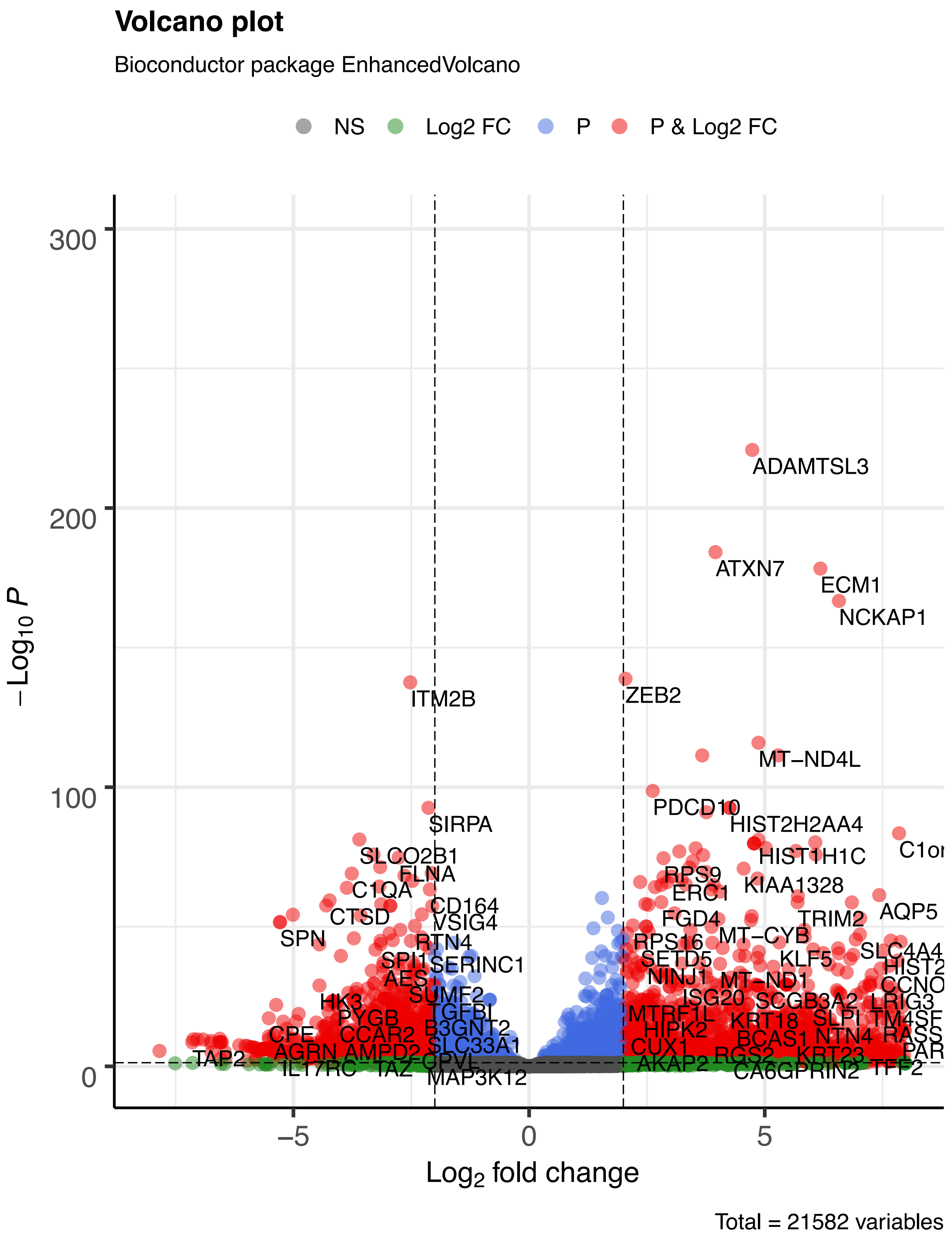

Figure S4. Volcano plot of BALF signature.

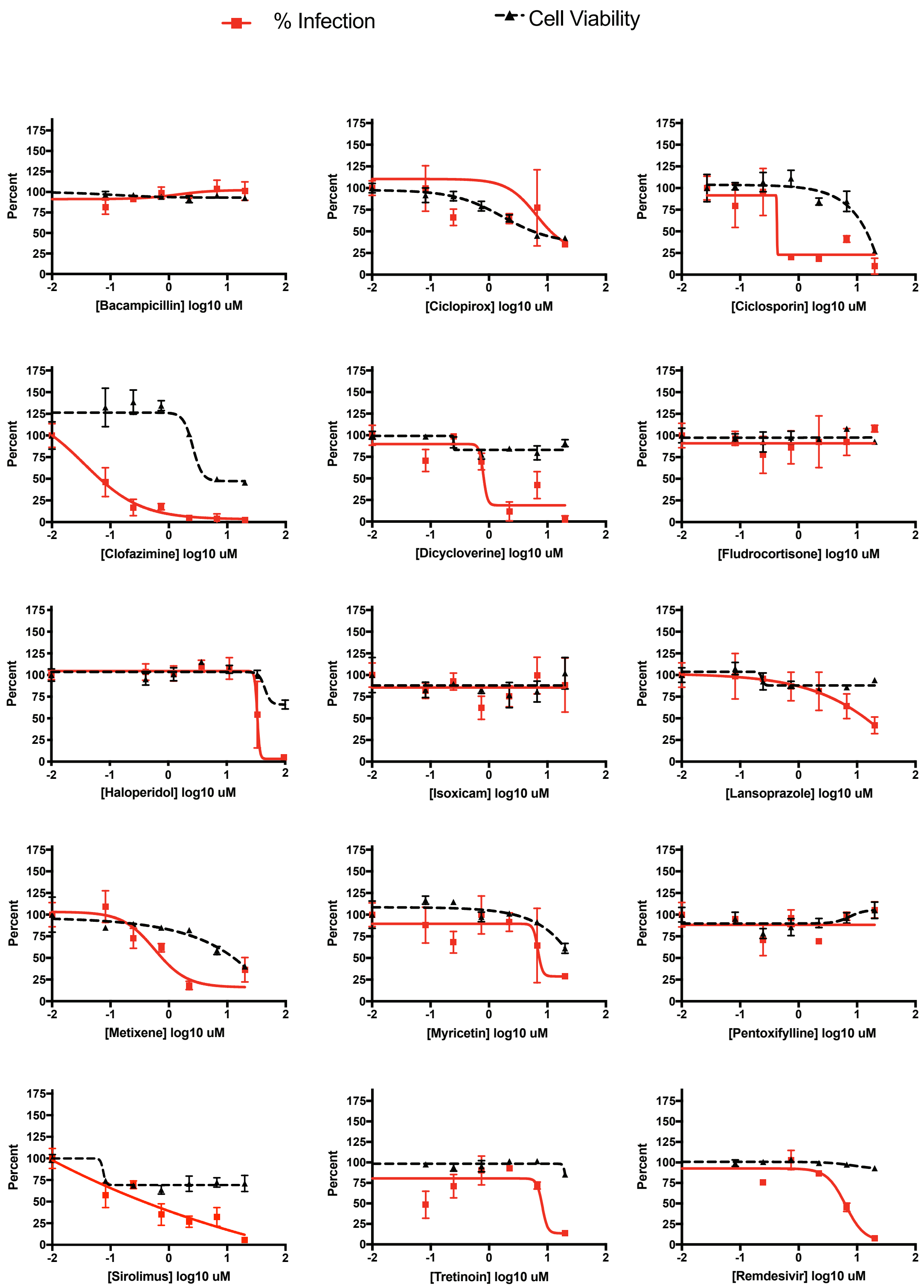

Figure S5. Vero E6 cell assays: Several compounds inhibit viral infectivity. Haloperidol was previously tested[12]. Red, viral infectivity (anti-NP); black, cell viability. The lack of a dose response in cell viability probably reflects cytostatic and not cytotoxic effects. Data are mean ± s.d.; *n* = 3 biologically independent samples for cell viability data.

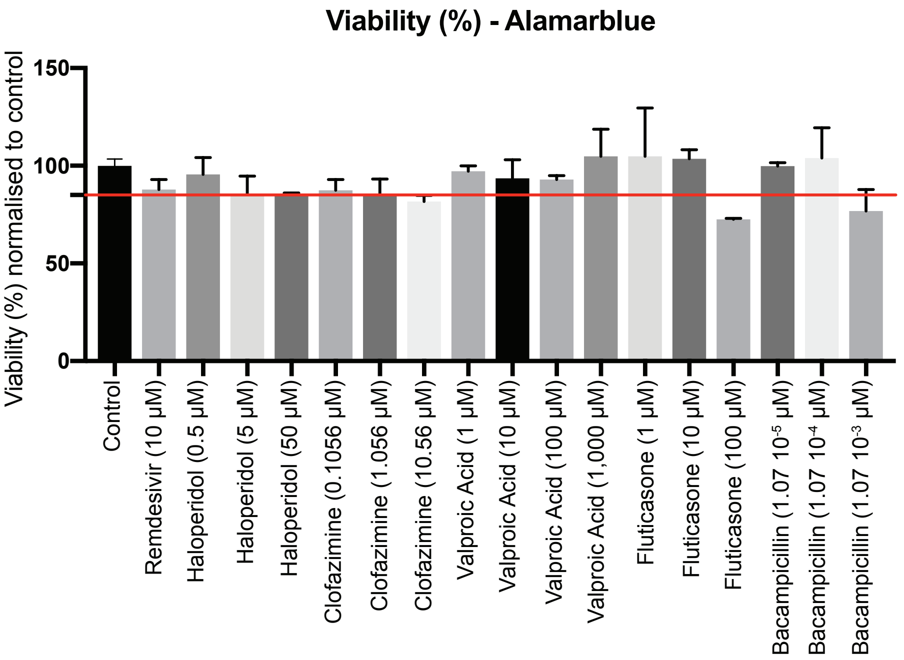

Dunn's multiple comparisons test	Mean rank diff.	Significant?	Summary	Adjusted P Value
Control vs. Remdesivir (10 ⌈M)	15	No	ns	>0.9999
Control vs. Haloperidol (0.5 ⌈M)	5.5	No	ns	>0.9999
Control vs. Haloperidol (5 ⌈M)	16.5	No	ns	>0.9999
Control vs. Haloperidol (50 ⌈M)	17	No	ns	>0.9999
Control vs. Clofazimine (0.05 ug/mL)	16.5	No	ns	>0.9999
Control vs. Clofazimine (0.5 ug/mL)	17.5	No	ns	>0.9999
Control vs. Clofazimine (5 ug/mL)	22	No	ns	0.6253
Control vs. Valproic Acid (1 uM)	3	No	ns	>0.9999
Control vs. Valproic Acid (10 uM)	8	No	ns	>0.9999
Control vs. Valproic Acid (100 uM)	8	No	ns	>0.9999
Control vs. Valproic Acid (1,000 uM)	0	No	ns	>0.9999
Control vs. Fluticasone (1 uM)	3.5	No	ns	>0.9999
Control vs. Fluticasone (10 uM)	-2.5	No	ns	>0.9999
Control vs. Fluticasone (100 uM)	26	No	ns	0.2311
Control vs. Bacampicillin (5 pg/mL)	0.5	No	ns	>0.9999
Control vs. Bacampicillin (50 pg/mL)	0.5	No	ns	>0.9999
Control vs. Bacampicillin (500 pg/mL)	23	No	ns	0.4935

Figure S6. Viability assay (Alamarblue) in Calu-3 cells treated with all the compounds for 72h (n=1, with 2 technical replicates).

Table S1. ALV signature – differentially expressed genes.

Table S2. EXP signature – differentially expressed genes.

Table S3. BALF signature – differentially expressed genes.

Table S4. 30 drug hits reversed the ALV signature.

Table S5. 15 drug hits reversed the EXP signature.

Table S6. 86 drug hits reversed the BALF signature.

Table S7. Drug hits – signatures reversed and targets. 102 unique drug hits were identified, with 25 drugs reversing at least 2 signatures. Target information was aggregated from DrugBank.
